# Autopercepción de calidad de vida en salud bucal de adultos de Valparaíso, Chile

**DOI:** 10.21142/2523-2754-1203-2024-205

**Published:** 2024-09-17

**Authors:** Alfredo Cueto, Fernanda Acevedo, Melanie Arnes, Daniela Carrasco, Arianna Gallardo, Patricia Reyes, Francisca Rodríguez, Nayeli Tavali

**Affiliations:** 1 , Facultad de Odontología, Universidad de Valparaíso, ValparaísoChile. alfredocuetourbina@yahoo.es Universidad de Valparaíso Facultad de Odontología Universidad de Valparaíso Valparaíso Chile alfredocuetourbina@yahoo.es; 2 Escuela de Pregrado. Facultad de Odontología, Universidad de Valparaíso. Valparaíso, Chile. fernanda.acevedo@alumnos.uv.cl, melanie.arnes@alumnos.uv.cl, daniela.carrascok@alumnos.uv.cl, arianna.gallardo@alumnos.uv.cl, patricia.reyes@alumnos.uv.cl, francisca.rodriguezma@alumnos.uv.cl, nayeli.tavali@alumnos.uv.cl Universidad de Valparaíso Escuela de Pregrado Facultad de Odontología Universidad de Valparaíso Valparaíso Chile fernanda.acevedo@alumnos.uv.cl melanie.arnes@alumnos.uv.cl daniela.carrascok@alumnos.uv.cl arianna.gallardo@alumnos.uv.cl patricia.reyes@alumnos.uv.cl francisca.rodriguezma@alumnos.uv.cl nayeli.tavali@alumnos.uv.cl

**Keywords:** salud oral, calidad de vida, autopercepción de la salud, oral health, quality of life, self-perception of health

## Abstract

**Objetivo:**

Caracterizar la autopercepción de la calidad de vida en salud bucodental de los adultos en la comuna de Valparaíso, Chile.

**Materiales y métodos:**

: Se realizó un estudio transversal tipo serie de casos en adultos residentes en la comuna de Valparaíso, con un muestreo por conveniencia. Se utilizó el instrumento GOHAI para medir la calidad de vida bucodental, aplicado en noviembre de 2023. Se llevó a cabo un análisis descriptivo de los datos y se evaluó la asociación entre la calidad de vida en salud bucal, medida mediante el puntaje del test GOHAI, y las variables independientes cualitativas.

**Resultados:**

La muestra estuvo compuesta por 113 encuestados, de los cuales el 60,18% fueron mujeres (n = 68) y el 39,82% fueron hombres (n = 45). La media de edad fue de 43,84 años. El GOHAI arrojó una media general de 47,33 puntos y una mediana de 49,00 puntos, indicando una buena calidad de vida. No se encontraron diferencias estadísticamente significativas entre los resultados totales de GOHAI con respecto a edad, sexo y el tipo de establecimiento de atención (público, privado, mixto; p > 0,05).

**Conclusiones:**

La calidad de vida con relación a la salud bucodental fue buena en el 73% de la muestra, mientras que el 27% se autoevaluó como regular o mala. La salud bucodental, cuando se ve negativamente afectada, repercute en la manera en que el individuo se relaciona con su entorno. Este porcentaje sugiere que las intervenciones en los pacientes del sistema público, privado y mixto aún no son suficientes para mejorar la calidad de vida bucodental de manera integral.

## Abstract (en)

**Objective:**

To characterize the self-perception of quality of life in oral health of adults in the commune of Valparaíso, Chile.

**Materials and methods:**

A cross-sectional case series study was carried out in adults residing in the commune of Valparaíso, with convenience sampling. The GOHAI instrument was used to measure oral quality of life, applied in November 2023. A descriptive analysis of the data was carried out and the association between quality of life in oral health, measured by the GOHAI test score, was evaluated, and the qualitative independent variables.

**Results:**

The sample was made up of 113 respondents, of which 60.18% were women (n=68) and 39.82% were men (n=45). The average age was 43.84 years. The GOHAI showed a general mean of 47.33 points and a median of 49.00 points, indicating a good quality of life. No statistically significant differences were found between the GOHAI total results with respect to age, sex, and type of care facility (public, private, mixed; p>0.05).

**Conclusions:**

Quality of life in relation to oral health was good in 73% of the sample, while 27% rated themselves as fair or poor. Oral health, when negatively affected, affects the way in which the individual relates to his or her environment. This percentage suggests that interventions in patients from the public, private and mixed system are still not enough to improve oral quality of life in a comprehensive manner.

## INTRODUCCIÓN

La salud bucodental constituye parte esencial de la vida del ser humano; sin los cuidados necesarios, puede verse afectada, lo que tiene como resultado, a veces, un cambio drástico en la vida de las personas. Los cambios que se producen en la salud bucodental a través del ciclo vital pueden involucrar aspectos estéticos, sangrado de encías, pérdida dentaria o caries, lo que puede afectar la manera como nos comunicamos e, incluso, una actividad tan simple y cotidiana como comer. La Federación Dental Internacional define que la salud bucodental “es polifacética e incluye, entre otros aspectos, la capacidad de hablar, sonreír, oler, saborear, tocar, masticar, tragar y transmitir una serie de emociones a través de las expresiones faciales con confianza y sin dolor, incomodidad ni enfermedad del complejo craneofacial” ^1^. A su vez, se menciona que la salud bucodental es un pilar importante en la salud, física y mental, que esta va a estar mediada por los valores, actitudes y costumbres de cada individuo y de las comunidades en las que se desarrollan, lo cual se reflejará en aspectos fundamentales como la calidad de vida, la cual será influenciada por percepciones de las personas, experiencias pasadas y expectativas de los pacientes, así como por su capacidad de cambio.

Calidad de vida, según la OMS, se define como “la percepción del individuo sobre su posición en la vida, en el contexto de la cultura y el sistema de valores en que vive, en relación con sus objetivos, expectativas, estándares y preocupaciones” ^2^. Este concepto ha evolucionado y abarca más aspectos de la vida humana, uno de ellos la salud bucodental, la cual se define como el bienestar físico, psicológico y social en relación con el estado bucodental. La autopercepción de calidad de vida, entendiéndose este primer concepto como “un conjunto de valores y pensamientos que tiene una persona de sí mismo en cuanto a sus acciones y su vida en un momento determinado, esto se ve influenciado por un conjunto de creencias, deseos, actitudes y expectativas que tiene la persona consigo mismo y con el mundo que lo rodea” ^3^.

La población adulta puede ver afectada su salud bucodental y con ello su calidad de vida. Estudios han demostrado una relación directa entre edad y prevalencia de caries ^4^. A su vez, la población adulta se puede ver afectada por enfermedades crónicas no transmisibles, como diabetes mellitus o hipertensión arterial ^5^, las cuales aumentan el riesgo de desarrollar una enfermedad periodontal y el riesgo cariogénico. Si estas enfermedades no son tratas a tiempo, pueden favorecer la pérdida dentaria a edad temprana, lo cual genera que pacientes adultos necesiten tratamiento rehabilitador con prótesis removible.

En 2006, en Chile, se realizó la Encuesta Nacional de Calidad de Vida y Salud, la cual dejó en evidencia que la salud bucal influye en la calidad de vida de la persona, esto en los ámbitos dolor, malestar, limitación, incluyendo una minusvalía social y funcional ^6^.

Si bien se han realizado estudios sobre la salud bucodental y el impacto sobre la calidad de vida en Chile ^7^^-^^10^, no han existido actualizaciones sobre la calidad de vida con relación a la salud bucal de los adultos desde 2016, por lo que no conocemos la percepción actual de las personas que puede verse afectada por los distintos cambios culturales, demográficos y epidemiológicos.

Por lo tanto, la pregunta central de esta investigación es: ¿Cuál es la percepción de la calidad de vida en salud bucal de los adultos que reciben atención primaria de salud bucodental en Valparaíso? El propósito del estudio es caracterizar la autopercepción de la calidad de vida en salud bucodental de los adultos en la comuna de Valparaíso, Chile.

## MATERIALES Y MÉTODOS

El protocolo de investigación fue aprobado por el Comité de Revisión de Proyectos de Investigación de la Facultad de Odontología de la Universidad de Valparaíso, mediante resolución PREG-07-23, que evaluó los aspectos éticos, entre ellos que el estudio no genere ningún riesgo para la salud de los encuestados, que la participación sea libre y voluntaria, el uso del consentimiento informado y el resguardo de los datos sensibles durante toda la investigación.

El diseño fue transversal, de tipo serie de casos, en adultos residentes en la comuna de Valparaíso. Se realizó un muestreo por conveniencia de adultos de la comuna durante noviembre de 2023. Los criterios de inclusión fueron adultos desde los 18 años y autovalentes. Se excluyó a quienes presentaran dificultades físicas o cognitivas que les impidieran responder de forma autónoma la encuesta.

Para medir la calidad de vida respecto de la salud bucodental, se usó el GOHAI ^11^^-^^13^, un instrumento de autorreporte compuesto por 12 preguntas que entregan un puntaje total entre 12 a 60 puntos, y que permiten definir tres categorías: 12-27, mala calidad; 28-43, calidad regular calidad; y 44-60, buena calidad. Los 12 ítems del instrumento abarcan tres dimensiones: 1) Función física, 2) Psicosocial, 3) Dolor e incomodidad. En primer lugar, la función física, incluyendo comer, hablar y tragar. En segundo lugar, la función psicológica, que incluye la preocupación o inquietud acerca de la salud oral, la insatisfacción con la apariencia, la autoconciencia sobre la salud oral y el evitar contacto con otras personas por problemas orales. Y en tercer lugar, el dolor e incomodidad. Los 12 ítems están en una escala Likert, cada uno con 5 respuestas punteadas según el siguiente detalle: “Siempre” (1 punto), “A menudo” (2 puntos), “A veces” (3 puntos), “Rara vez” (4 puntos) y “Nunca” (5 puntos). 

El instrumento GOHAI ha sido ampliamente validado. En Colombia, se verificó su consistencia interna a través de alfa de Cronbach, con valores entre 0,87, en Cartagena ^11^, y 0,80 en Bogotá ^12^, lo que señala que es un instrumento confiable, que puede ser utilizado tanto en población geriátrica como adulta. Chile ^13^ validó el instrumento y obtuvo un valor de correlación test retest de 0,955, de lo cual se concluye su fiabilidad. Además, en este estudio se recogieron las siguientes variables: edad, sexo y centro de atención en una escala de público, privado o mixto, es decir, que acuden a ambos establecimientos de salud. 

El análisis de los datos obtenidos se realizó por etapas, comenzando con un análisis univariante de las variables. Para las variables cualitativas correspondientes a sexo (femenino, masculino) y establecimiento de atención en salud (público, privado y mixto), se realizaron tablas de frecuencia absoluta, frecuencia relativa y frecuencia relativa acumulada. Por otra parte, para las variables cuantitativas, como edad, funcionalidad, impacto psicológico, dolor o incomodidad, se realizaron estadísticas descriptivas. Además, la edad fue categorizada en rangos con base en el ciclo vital individual, agrupándose en “Adolescentes y Adulto joven”, desde los 18 años hasta los 39 años; “Adulto maduro”, desde los 40 a 64 años, y “Adulto mayor”, sobre 65 años.

Luego, se realizó un análisis bivariado con una prueba de Chi cuadrado para evaluar si existe una asociación significativa entre la calidad de vida en salud bucal y las variables independientes: sexo, establecimiento que frecuenta y edad. Se evaluó la asociación entre la calidad de vida en salud bucal en puntaje del test Gohai y las variables independientes cualitativas, por medio de gráficos de *box plot*.

## RESULTADOS

La muestra estuvo compuesta por 113 encuestados, de los cuales 60,18% fueron mujeres (n = 68) y 39,82% fueron hombres (n = 45), con edades entre 18 y 87 años. La media de la edad fue de 43,84 años, con un intervalo de confianza del 95% entre 39,99 y 47,01 años (desviación estándar de 20,65).

Respecto de los resultados del instrumento GOHAI, estos tienen un promedio general de 47,33 puntos; una mediana de 49,00 puntos, es decir, buena calidad de vida; y un rango entre 22,00 y 60,00 puntos. Los resultados por dimensión fueron los siguientes: funcionalidad presenta un promedio general de 16,82 puntos, una mediana de 18 puntos y un rango entre 7 y 20 puntos; incomodidad tiene un promedio general de 11,62 puntos, una mediana de 12 puntos y un rango entre 6 y 15 puntos; e impacto psicológico tiene un promedio general de 18,88 puntos, una mediana de 20 puntos y un rango entre 5 y 25 puntos.

Los participantes, según ciclo de vida, se distribuyeron en “Adolescente y adulto joven” (18-39 años) (n = 59 y 52,21%), “Adulto medio” (40-64 años) (n = 30 y 26,55%) y “Adulto mayor” (≥ 65 años) (n = 24 y 21,24%). El grupo “Adolescente y Adulto joven” obtuvo una media de 48,73 puntos, seguido por aquellos que pertenecieron al ciclo “Adulto medio”, con 46,73 puntos y, finalmente, el grupo “Adulto mayor”, con una media de 44,67 puntos, sin diferencias estadísticamente significativas entre grupos. En promedio, cada uno informa buena calidad de vida relacionada con la salud bucodental.

En los grupos “Adulto medio” (n = 30) y “Adulto mayor” (n = 24) se presentan los únicos casos que reportan una mala calidad de vida respecto de su salud bucodental, lo que representa un 3,33% (n = 1) y un 8,33% (n = 2), respectivamente.

Con respecto a los establecimientos de salud que frecuentan los participantes, un 41,59% (n = 47) asiste a establecimientos públicos; un 30,97% (n = 35), a establecimientos mixtos; y un 27,43% (n = 31), a establecimientos privados. Tanto los que asisten a establecimientos privados, con una media de 49,10 puntos, como aquellos que frecuentan establecimientos públicos, con 48,13 puntos, y los que asisten a establecimientos mixtos, con 45,16 puntos, informan una buena calidad de vida y, en consecuencia, no se presentan entre ellos diferencias estadísticamente significativas.

En cuanto a la percepción de calidad de vida en salud bucodental entre sexo femenino y masculino, las participantes de sexo femenino (n = 68) declararon una autopercepción en torno a calidad de vida en salud bucal con una media de 47,76 puntos, semejante a los participantes de sexo masculino (n = 45), quienes obtuvieron una media de 47,06 puntos.

Al respecto, un 73,53% (n = 50) de las participantes de sexo femenino declaró tener una buena calidad de vida en salud bucal; un 23,53% (n = 16), una calidad de vida regular; y un 2,94 (n = 2), una mala calidad de vida. Por otra parte, un 73,33% (n = 33) de los participantes de sexo masculino manifestó presentar una buena calidad de vida con relación a su salud bucal, un 24,44% (n = 11), una calidad de vida regular; y un 2,22% (n = 1), una mala calidad de vida (ver Figura 2).

**Figura 1:**
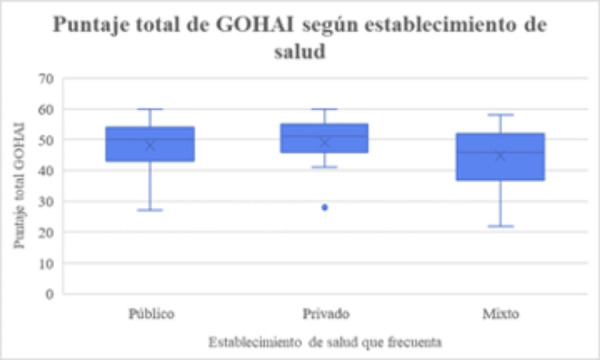
Puntaje total de GOHAI de acuerdo a establecimientos de salud que frecuenta una muestra de población de adultos de la comuna de Valparaíso.

**Figura 2:**
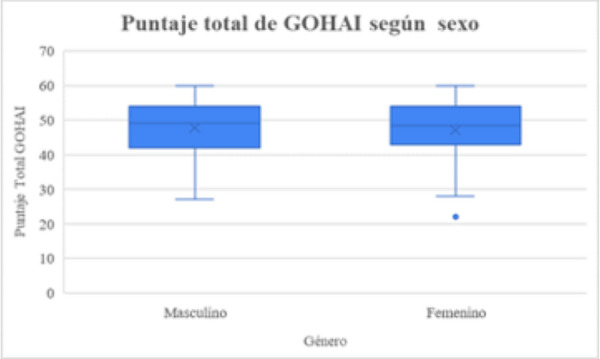
Puntaje total de GOHAI de acuerdo a sexo de una muestra de población de adultos de la comuna de Valparaíso.

No se encontraron diferencias estadísticamente significativas entre los resultados totales de GOHAI y edad, sexo y establecimientos que frecuentan los participantes (p > 0,05) (ver Figuras 1, 2 y Tabla 1).

**Tabla 1:** Media (DE: desviación estándar) y análisis bivariado del puntaje de GOHAI de acuerdo con las características de una muestra de población de adultos de la comuna de Valparaíso

					GOHAI							
					Buena calidad		Regular calidad		Mala calidad		
Variable	Categorías	n	%	Media (DE)	n	%	n	%	n	%	Valor de p
Edad	Adolescente y adulto joven	59	52,21	48,73 (6,83)	46	77,97	13	22,03	0	0	0,24
	Adulto medio	30	26,55	46,73 (9,48)	22	73,33	7	23,33	1	3,33	
	Adulto mayor	24	21,24	44,67 (10,71)	15	62,5	7	29,17	2	8,33	
Sexo	Hombre	45	39,82	47,76 (8,52)	33	73,33	11	24,44	1	2,22	1
	Mujer	68	60,18	47,06 (8,67)	50	73,53	16	23,53	2	2,94	
Establecimientos de salud	Público	47	41,59	48,13 (7,7)	34	72,34	12	25,53	1	2,13	0,4
	Privado	31	27,43	49,1 (8,37)	26	83,87	5	16,13	0	0	
	Mixto	35	30,97	45,16 (9,8)	23	65,71	10	28,57	2	5,71	

DE: Desviación estándar; n: frecuencia; %: porcentaje de n

## DISCUSIÓN

En uno de los últimos estudios llevados a cabo en Chile, entre los años 2015 y 2016, respecto de la calidad de vida relacionada con salud oral y la autopercepción de salud, se llegó a la conclusión, a través del instrumento Oral Health Impact Profile (OHIP-7), de que un 18,50% de la población percibe que sus problemas de salud oral impactan en su calidad de vida. En esta publicación, los factores a los que se asoció una mala percepción de calidad de vida relacionada con la salud oral fueron una mala autopercepción de salud general, la edad (45-54 años) y el uso frecuente de medicamentos. De igual forma, otro estudio llegó a una conclusión similar: “Siendo más fuerte esta peor calidad de vida en salud oral a medida que avanza la edad y con el uso frecuente de medicamentos” ^14^. En nuestro estudio, 30 personas (26,5%) consideran regular o mala su calidad de vida en salud bucodental, es decir, que sus problemas de salud oral impactan en su calidad de vida, lo que implica un mayor porcentaje que el registrado a nivel nacional en 2016. Esto se puede explicar porque se entrevistó en sectores populares de la ciudad; en consecuencia, pueden tener mayores necesidades de salud que la población promedio.

El nivel socioeconómico se ha relacionado directamente con el acceso a los servicios de salud y la capacidad de las diferentes personas de acceder a una cobertura odontológica de calidad ^15^; sin embargo, en los resultados de este estudio, no se halló diferencias entre sistema público, privado y mixto. Esto se puede atribuir a varias razones. Por un lado, si bien es cierto que la cobertura pública es baja, la calidad de la atención es percibida como adecuada, en especial por los beneficiarios de Garantías Explícitas en Salud ^16^. Por otro lado, la disponibilidad de oferta privada de odontología en Chile ha aumentado ^17^; no obstante, los costos o precios de acceso siguen siendo prohibitivos para amplios sectores, los que solo acceden a prestaciones asistenciales, en vez de las indicadas según el caso clínico, lo que genera disconformidad. 

Entre las dimensiones que registra el GOHAI, la incomodidad presentó el menor puntaje, categorizada como regular. Por la edad de los participantes, es probable que un grupo numeroso use aparatología removible, la cual está asociada con mayores molestias por desajustes, lesiones irritativas de los tejidos, reabsorción de hueso alveolar, etc., lo que dificulta funciones como comer, hablar y sonreír ^18^.

### Limitaciones

Entre las limitaciones del estudio está el trabajar con una muestra por conveniencia, pues no necesariamente representa a la población; sin embargo, se atenuó con un tamaño que permitió el análisis estadístico. Por otra parte, debieron haberse recogido otras variables que pudieran explicar el fenómeno de estudio, como nivel socioeconómico y uso de medicamentos. Una limitación inherente a las encuestas de autorreporte es que no garantizan que todos los sujetos sean capaces de responder un cuestionario.

## CONCLUSIONES

La calidad de vida con relación a la salud bucodental, medida por medio del GOHAI, muestra una buena calidad en el 73% de la muestra. No resultaron variables explicativas la edad, el sexo ni el establecimiento de atención.

El 27% se autoevaluaron con regular y mala calidad de vida respecto de la salud bucodental. Estos resultados son menores a los de 2006 (37%) y mayores a los de 2016 (18,5%), en estudios a nivel nacional. Estas diferencias probablemente correspondan a los tipos de muestreo, pero son consistentes y persistentes para demostrar las necesidades de salud bucodental en la población.

Deberían implementarse medidas para mejorar el impacto de la salud bucodental en la calidad de vida de las personas, como la creación de políticas públicas que abarquen no solo los microdeterminantes en salud, sino que se orienten a los macrodeterminantes, para así abarcar factores de riesgo que en la actualidad perjudican la vida de los adultos.

## Financiamiento:

Estudio autofinanciado
